# Influence of Age at Harvest and Packaging Conditions on Color Stability of Bovine *Psoas major* Muscle

**DOI:** 10.3390/foods14132197

**Published:** 2025-06-23

**Authors:** Xiao Lu, Xin Luo, Sulaiman K. Matarneh

**Affiliations:** 1 Department of Nutrition, Dietetics and Food Science, Utah State University, Logan, UT 84322, USA; waitingfor90@163.com; 2 College of Food Science and Engineering, Shandong Agricultural University, Taian 271018, China

**Keywords:** beef, *Psoas major*, age at harvest, packaging conditions, color stability

## Abstract

This study investigated the effect of cattle age on the color stability of *Psoas major* steaks under different packaging conditions, including polyvinyl chloride (PVC) film packaging, high-oxygen-modified atmosphere packaging (HiOx-MAP), and carbon monoxide-modified atmosphere packaging (CO-MAP), over a 14-day storage period. Steaks from old cows had a greater myoglobin content than those from young animals (*p* < 0.05), resulting in a darker beef color. Regardless of animal age, steaks in PVC and HiOx-MAP exhibited poor color stability during storage, as evidenced by the low color intensity and high surface metmyoglobin (MetMb; *p* < 0.05). These samples also exhibited a reduced MetMb reducing activity (MRA) and oxygen consumption rate (OCR) and higher 2-thiobarbituric acid reactive substance (TBARS) levels, with these effects being evident in steaks from mature cows. In contrast, steaks stored in CO-MAP had greater redness, MRA, and OCR, along with lower surface MetMb and TBARS levels (*p* < 0.05), contributing to the limited discoloration of steaks from both age groups. These findings suggest that while both harvest age and packaging affect the shelf stability of bovine steaks, packaging plays a more significant role. CO-MAP proved to be more effective than aerobic packaging and may mitigate age-related differences in color stability.

## 1. Introduction

Of all the quality traits affecting purchasing decisions of fresh meat, color is one of the most significant [1]. Meat color is usually perceived as an indicator of freshness and wholesomeness by consumers at the point of sale [2]. Age at harvest is one of the intrinsic factors influencing meat color. Older animals have been shown to possess a higher myoglobin concentration than their younger counterparts [3,4,5], so meat from older animals is associated with a darker color. Several studies have outlined the negative effect of increasing harvest age on beef color. Muramoto et al. [6] observed that the color stability of Japanese Black steers became poor with the increase in harvest age. Similarly, Cho et al. [5] found that meat from older Korean Hanwoo cows was darker and had lower color stability in comparison to that of younger animals. However, the relationship between slaughter age and beef color is still controversial. Galli et al. [7] reported that age at harvest did not significantly affect the lightness (L*), redness (a*), and yellowness (b*) values of striploin steaks from Hereford cows. Pflanzer and de Felício [8] also concluded that carcass maturity did not affect the surface color of rib steaks from Nellore steers. Therefore, further research is needed to better understand the effect of harvest age on the color stability of beef muscle during storage.

The choice of packaging method significantly affects the surface color, color stability, and microbial shelf life. Steaks in styrofoam trays overwrapped with oxygen-permeable polyvinyl chloride (PVC) films are initially bright red, but discoloration occurs rapidly. Accelerated discoloration in high oxygen environments is typically attributed to greater lipid oxidation, which subsequently promotes myoglobin oxidation [9]. Compared to PVC packaging, fresh meat in high-oxygen-modified atmosphere packaging (HiOx-MAP) or carbon monoxide-modified atmosphere packaging (CO-MAP) can maintain the attractive red color with greater color stability and a longer shelf life [10,11,12].

The *Psoas major* muscle is of interest because of its inherent tenderness. It is a primary muscle from mature cow carcasses that has retail value as a steak rather than being processed into jerky or comminuted meat. Bovine *Psoas major* is well known to develop discoloration very quickly; however, the effects of harvest age and packaging conditions on its color stability are poorly understood. Therefore, the objective of this study was to investigate the effect of slaughter age and packaging type on the color stability of bovine *Psoas major* muscle.

## 2. Materials and Methods

### 2.1. Muscle Samples

*Psoas major* muscles from both sides of four young Holstein cattle (22–24 months old, USDA select grade) and four mature Holstein cows (48–60 months old, ungraded cull cows) were obtained on day 5 postmortem from a commercial abattoir. Cattle in each age category had similar genetics and were raised under uniform feeding and management practices. All the muscles had a normal ultimate pH (pHu = 5.55–5.65) and were individually vacuum packaged and transported on ice to the Meat Research Laboratory at Utah State University.

### 2.2. Packaging and Storage

*Psoas major* muscles from each animal were portioned into 2 cm thick steaks (13 steaks/animal, 104 steaks in total). One steak from each animal without any packaging was used for measurements on day 0. The remaining 12 steaks from each animal were randomly assigned to one of the following treatments (4 steaks per treatment): PVC (styrofoam trays overwrapped with PVC film), HiOx-MAP (80% O_2_, 20% CO_2_), or CO-MAP (0.4% CO, 30% CO_2_, 69.6% N_2_). The packaging machine (Promax CE1, Promax Packaging, Ontario, CA, USA) was capable of either vacuum packaging or gas flushing and sealing of modified atmosphere packages. The gas cylinders for MAP were obtained from Praxair Distribution (Salt Lake City, UT, USA) and certified to be within ±0.5% of the indicated mixtures. The PVC film used in this study (AEP Industries Inc., South Hackensack, NJ, USA) had an oxygen transmission rate of 98.4 cm^3^/m^2^/24 h at 23 °C and a water vapor transmission rate of 496 g/m^2^/24 h at 37.8 °C and 90% relative humidity. The pouches (20 × 30 cm; LK Plastics, Los Angeles, CA, USA) used for HiOx-MAP and CO-MAP were a two-layer film consisting of an inner polyethylene layer and an outer cast nylon layer. They were 76 µm thick, with an oxygen permeability of 0.6 cm^3^/m^2^/24 h at 0 °C and a water vapor transmission rate of 0.6 g/m^2^/24 h at 38 °C and 100% relative humidity. All the steaks were individually packaged and labeled and then stored in a cooler at 4 °C for 14 days. For each animal at each age group and packaging method, single samples were removed for analysis at 4, 7, 10, and 14 days of storage.

### 2.3. Total Myoglobin Content

The myoglobin content was analyzed as described by the AMSA [13] with minor modifications. Five grams of chopped samples was mixed with 25 mL of ice-cold phosphate buffer (pH 6.8, 0.04 M) and homogenized for 40 s at low speed (Ultra-Turrax T18 homogenizer, T18 Basic; IKA, Staufen, Germany). The homogenates were held on ice for 1 h before being centrifuged at 3000× *g* for 30 min at 4 °C. The supernatant was then filtered through Whatman #1 filter paper (Whatman Cytiva, Marlborough, MA, USA), and the absorbance at 525 nm was measured spectrophotometrically. The total myoglobin content was then calculated by the following equation: Myoglobin concentration (mg/g meat) = (A525/7.6) × 17 × 6.

### 2.4. Color Measurement

The surface meat color was measured immediately after the packages were opened, using a Hunter lab Miniscan portable colorimeter (Reston, VA, USA) with illuminant D65, a 10° observer angle, and an 8 mm aperture diameter. The instrument was calibrated with white and black standard plates before measurements. Each sample was measured at 3 different locations. The color coordinate (L*, a*, and b*) values were recorded, and the hue angle (h*) was calculated using the equation: h* = arc tan (b*/a*). In addition, the reflectance values were also recorded in the range of 400 to 700 nm at 10 nm intervals, and the Kubelka–Munk K/S values were then calculated. The content of metmyoglobin (MetMb) on the meat surface was estimated by the K/S 572/525 ratio according to the AMSA [13].

### 2.5. Metmyoglobin Reducing Activity

The MetMb reducing activity (MRA) was determined according to the procedure of Sammel et al. [14] with minor modifications. A cube (2.54 × 2.54 × 2 cm) with no visible fat or connective tissue was removed from each steak. The cube was then bisected parallel to the steak surface, resulting in 2 pieces. The top piece, exposed to the atmosphere of the package, was used to measure the MRA. This piece was oxidized in 50 mL of 0.3% (wt/vol) sodium nitrite solution for 20 min at room temperature and then removed, blotted dry, vacuum packaged, and immediately scanned three times using the colorimeter mentioned above to calculate the initial MetMb%. Subsequently, the sample was placed in an incubator at 30 °C for 2 h and rescanned to calculate the final MetMb%. The MRA was calculated as the percent of MetMb reduced: [(Initial MetMb% − Final MetMb%)/Initial MetMb%] × 100.

### 2.6. Oxygen Consumption Rate

The fresh-cut surface of the bottom piece from the cube mentioned above (originally in the center of the cube) was used to measure the oxygen consumption rate (OCR) according to a modified procedure based on Madhavi and Carpenter’s method [15]. Samples were allowed to bloom and then were vacuum packaged and scanned three times on the bloomed surface after packaging to measure the initial oxymyoglobin percentage (OxyMb%). After incubation at 30 °C for 30 min, samples were rescanned to determine the final OxyMb%. The OCR was calculated as the percent of OxyMb consumed: [(initial OxyMb% − Final OxyMb%)/Initial OxyMb%] × 100.

### 2.7. Lipid Oxidation

Lipid oxidation was determined by the 2-thiobarbituric acid reactive substances (TBARS) method according to the procedure by Lu et al. [16]. Samples were trimmed to a depth of 3 mm from the steak surface. A portion of trim (2.5 g, without visible fat or connective tissue) was homogenized with 12.5 mL of distilled water for 1 min. Then, 12.5 mL of 10% (*w*/*v*) trichloroacetic acid was added, the sample was vortexed, and it was filtered through Whatman #1 filter paper. The filtrate (~4 mL) was obtained and added to a centrifugal tube with 1 mL of 0.06 M TBA. The mixture was incubated in a water bath at 80 °C for 90 min, and the absorbance at 532 nm was then measured spectrophotometrically (UV-2600, Shimadzu, Kyoto, Japan), while the blank was set as the mixture of 2 mL of distilled water + 2 mL of 10% trichloroacetic acid + 1 mL of 0.06 M TBA. The results are expressed in mg malondialdehyde (MDA)/kg sample.

### 2.8. Red Color Penetration Depth

The carbon monoxide and oxygen penetration depth were measured on cross-sections of each steak. Vertical cuts, perpendicular to the steak surface, were made to view the sub-surface cross-section. Then, measurements of the depth of bright red color penetration were taken in mm with a digital caliper. The penetration depth values were the mean of three measurements on a single slice of the sample.

### 2.9. Statistical Analysis

The total myoglobin content measurement was performed using the 0-day sample only. As such, total myoglobin was analyzed by one-way analysis of variance using the general linear model procedure of SAS (version 9.2, SAS Institute, Inc., Cary, NC, USA). For all other parameters, the MIXED procedure of SAS was used with slaughter age, storage duration, packaging treatments, and their interactions fitted as fixed effects and animal as a random effect. Tests of differences between predicted means were applied using the PDIFF statement, and differences were considered significant at *p* < 0.05. Data are least-squares means ± pooled SE.

## 3. Results and Discussion

### 3.1. Total Myoglobin Content

**Figure 1:**
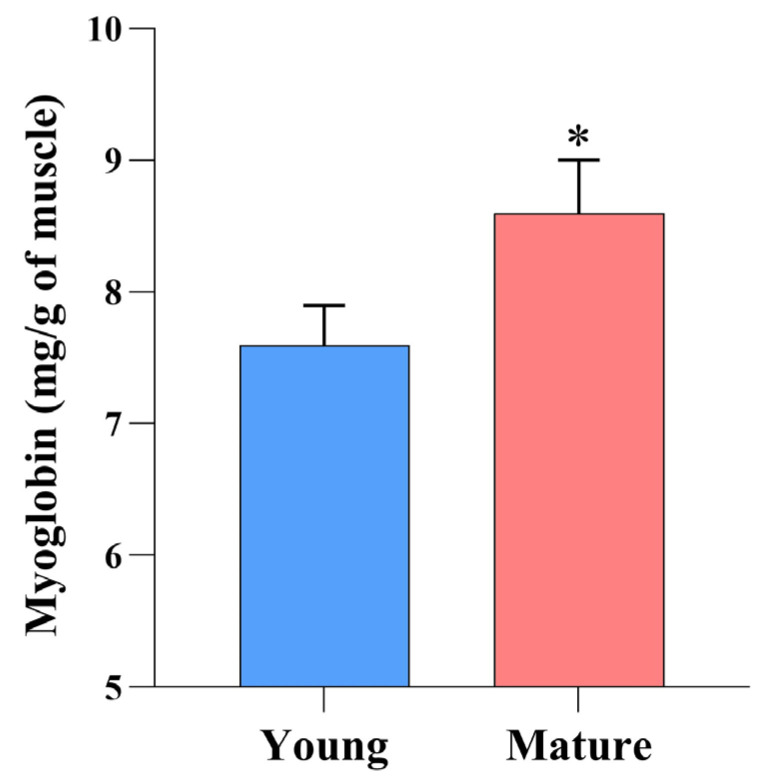
Effect of animal age on the myoglobin content of bovine *Psoas major* steaks. * Indicates significant difference (*p* < 0.05).

The myoglobin content is the determining factor influencing meat color, and the redox forms of myoglobin are susceptible to packaging conditions [17]. In this study, the total myoglobin content in the muscle was significantly affected by animal age (Figure 1). Mature cows had a greater myoglobin concentration compared to young animals (*p* < 0.05), which was consistent with other findings that myoglobin levels increase as animals become older [3,4,5]. The increase in myoglobin concentration is also supported by Purchas and Busboom [18], who reported a greater accumulation of iron in muscles from old cattle.

### 3.2. Surface Color

Animal age, packaging method, storage time, and their interactions had significant effects on the L*, a*, and h* values (*p* < 0.05). As expected, steaks from mature cows had lower initial L* values compared to young animals (Table 1), reflecting the higher myoglobin content seen in older animals (Figure 1). As the storage time was extended, the L* value of PVC-packaged steaks remained relatively stable, at about 33.5 and 30.5 for the young and mature animal groups, respectively. In contrast, steaks in HiOx-MAP and CO-MAP from both the young and mature animals showed higher L* values than those in PVC at 4, 7, 10, and 14 days. These results align with the findings of Kim et al. [3], who similarly reported higher L* values for lamb stored under HiOx-MAP compared to PVC. Notably, steaks from mature cows in CO-MAP animals were darker than those in HiOx-MAP on day 4 (36.7 and 38.7, respectively), although this difference disappeared by 7 days of storage.

At day 0, both age groups developed a noticeable bloom, with a* value of 13.7 for young cows and 16.1 for mature cows. For PVC-packaged steaks from young animals, a* remained constant until day 4 and then dropped significantly to 7.8 by day 14, accompanied by extensive browning. PVC-packaged steaks from mature cows had a greater a* value at day 0 but were less color stable, with a* dropping from 16.1 on day 0 to 7.2 on day 14. Steaks from young animals in HiOx-MAP maintained the bloom from day 0 until day 4, with the a* value increasing from 13.7 to 17.3 before declining to 14.6 on storage day 14. However, HiOx-MAP did not improve color stability for steaks from mature animals, as a* declined from an initial value of 16.1 to 10.1 on day 14. Browning in aerobic packaging (PVC and HiOx-MAP) could be associated with increased lipid oxidation that induced myoglobin oxidation, forming more metmyoglobin on the surface of the meat [19] and thus reducing a*. In contrast to aerobic packaging systems, CO-MAP significantly preserved the a* value of steaks from both age groups throughout storage. Steaks from young animals in CO-MAP had a relatively low a* value of 13.7 on day 0, but it gradually increased to 17.9 on day 14, with visibly increasing redness. Steaks from mature cows in CO-MAP increased in redness from 16.1 on day 0 to 19.9 on day 14. Holman et al. [20] reported that beef color was considered acceptable when the a* values were equal to or above 14.5. After 7 days of storage, only steaks in CO-MAP had an acceptable red appearance without evidence of browning. Steaks from mature cows held under CO-MAP maintained higher a* values than those of CO-MAP steaks from younger cattle, probably due to the differences in the myoglobin content within age groups.

The main effects of packaging method and storage time, but not animal age, affected the b* values. The yellowness values increased from 13.1 on day 0 to 13.6 or 15.5 on day 4 for steaks from young animals in PVC or HiOx-MAP, respectively. However, the b* values of steaks from young cattle in anaerobic CO-MAP tended to decrease over the entire storage period. The yellowness values of steaks from mature cows exhibited a downward trend, irrespective of the packaging methods.

Larger h* values indicate less red color and more yellow color [21]. In the present study, steaks from both young and mature animals in PVC packaging exhibited the most pronounced increase in h* values, which indicates that PVC-packaged steaks had the lowest color stability. No significant changes occurred for the h* values of steaks from both age groups in HiOx-MAP, except for a dramatic increase for steaks from mature cows at the end of the storage period. Steaks in CO-MAP had the lowest h* values in this study, which agreed with Jayasingh et al. [22], who observed lower h* values for 0.5% CO-MAP steaks compared with PVC steaks.

In summary, steaks from mature cows were initially darker than those from young animals. While HiOx-MAP and CO-MAP maintained higher L* values over time compared to PVC, only CO-MAP effectively maintained a* in both age groups during storage. In contrast, PVC and HiOx-MAP were less effective, particularly for mature cow steaks, exhibiting sharp declines in a*. These results highlight CO-MAP’s superior ability to preserve visual meat quality, which is crucial for extending the shelf stability of meat.

### 3.3. Surface Metmyoglobin Content

**Figure 2:**
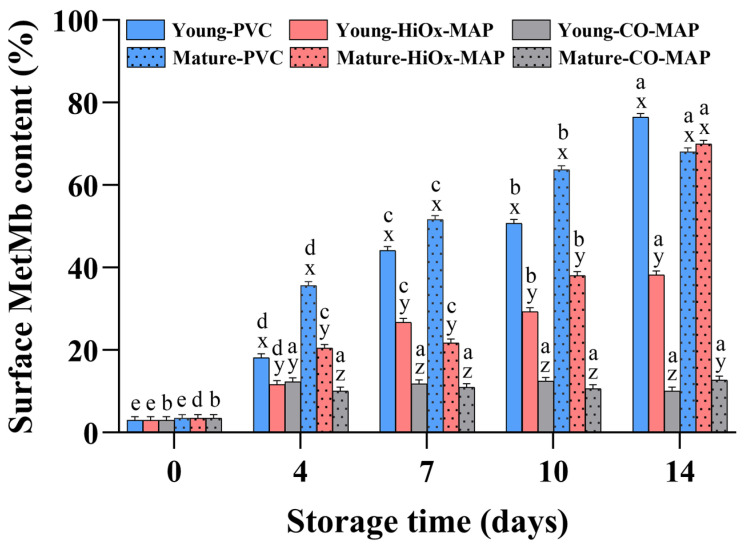
Effect of animal age, packaging method, and storage time on the surface content of MetMb (metmyoglobin) of bovine *Psoas major* steaks. Different letters (a–e) indicate significant differences between storage time points for the same age group and packaging method; different letters (x–z) indicate significant differences between packaging methods for the same age group and storage time point (*p* < 0.05).

The results of the surface MetMb content in young and mature cows under different packaging systems are presented in Figure 2. There were significant effects of slaughter age, packaging method, storage time, and their interactions on MetMb formation (*p* < 0.05), but no significant difference was found for the initial MetMb content between different age groups. The MetMb content continuously increased in PVC and HiOx-MAP during the entire storage period. Greene et al. [23] reported that when MetMb constitutes more than 40% of the total pigments on the surface, the appearance becomes brown, and consumers make a no-purchase decision. Steaks in PVC had more than 40% surface MetMb after 7 days of storage, with noticeable visual discoloration at day 14. It was noteworthy that steaks from mature cows packaged in HiOx-MAP exhibited more than 70% MetMb content on day 14, indicating severe discoloration. Mature cows showed distinctly higher MetMb formation than younger cattle in PVC and HiOx-MAP, but not in CO-MAP, likely because the anaerobic atmosphere of CO-MAP inhibited MetMb formation. However, the MetMb content increased moderately in the first 4 days under CO-MAP, which was likely due to a small amount of residual oxygen [11,12,24]. Girard et al. [25] similarly demonstrated that the MetMb content was significantly higher in steaks from yearling-fed steers (18–20 months old) than from calf-fed steers (12–13 months old). This difference was proposed to be related to the different feeding systems [26]. All in all, MetMb accumulation increased over storage, especially in PVC and HiOx-MAP, leading to discoloration. Steaks from mature cows showed higher MetMb levels than those from younger ones, except under CO-MAP, which effectively limited oxidation. These results highlight CO-MAP’s superiority in reducing discoloration and maintaining visual appeal.

### 3.4. Metmyoglobin Reducing Activity

**Figure 3:**
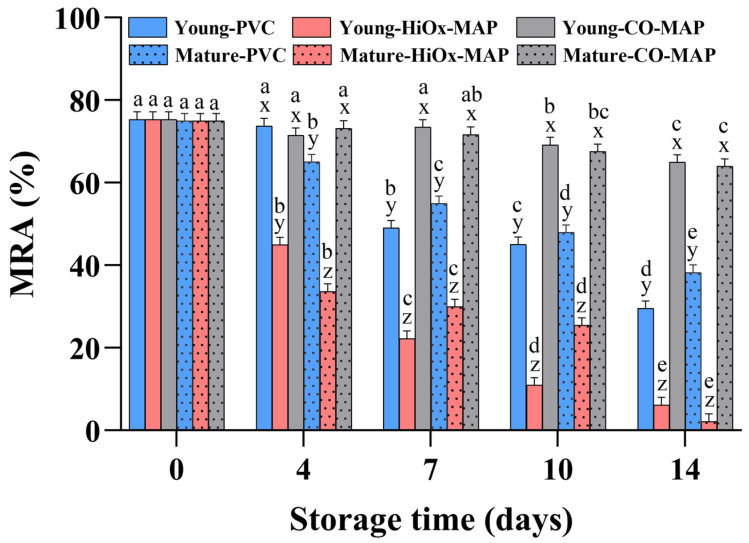
Effect of animal age, packaging method, and storage time on the metmyoglobin reducing activity (MRA) of bovine *Psoas major* steaks. Different letters (a–e) indicate significant differences between storage time points for the same age group and packaging method; different letters (x–z) indicate significant differences between packaging methods for the same age group and storage time point (*p* < 0.05).

The MRA indicates the ability of meat to convert MetMb back to deoxymyoglobin [27]. A higher MRA is associated with a greater inherent ability to reduce metmyoglobin in muscles, thereby improving meat color stability during storage [28]. Animal age, packaging method, storage time, and their interactions significantly affected the MRA in the present study (*p* < 0.05), but no significant difference was found for the initial MRA between different harvest age groups (Figure 3). The MRA values of both the young and mature cow groups significantly decreased during storage. Steaks from young cattle maintained a higher MRA value compared to those of mature animals at 4 days, particularly in the aerobic packages. CO-MAP steaks maintained a relatively high MRA value throughout the storage time compared to PVC and HiOx-MAP.

In summary, the MRA results were affected by relative oxidative stress, with lower MRA values observed in steaks in aerobic packaging, especially at longer storage time points. Zhang et al. [24] and Yang et al. [29] reported similar MRA results in bovine Longissimus muscle. Steaks in CO-MAP had a higher MRA than those in PVC and HiOx-MAP in this study, perhaps due to the anaerobic environment in CO-MAP protecting enzyme-based MetMb reducing systems from damage by O_2_-induced lipid oxidation products [30]. Steaks from mature cows had a lower MRA than steaks from young cattle during the first 4 days of storage, possibly because of a less active MetMb reductase enzyme system or less efficient replenishment of NADH or other biological reductants in steaks from older animals [31].

### 3.5. Oxygen Consumption Rate

**Figure 4:**
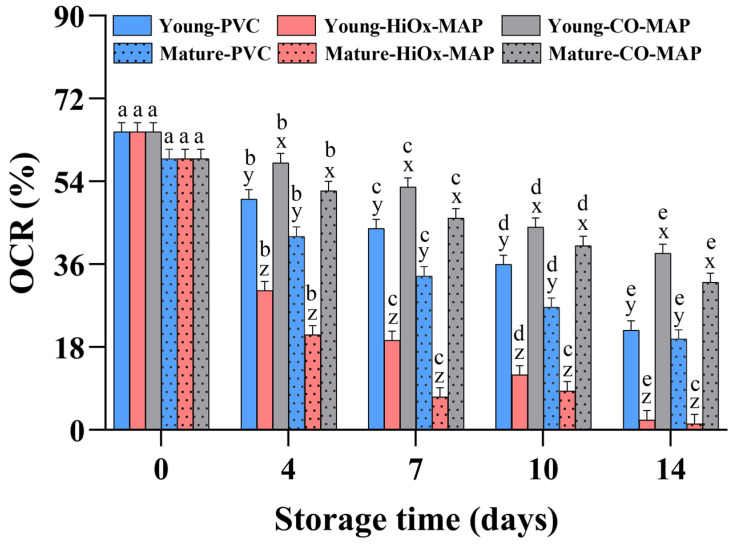
Effect of animal age, packaging method, and storage time on the oxygen consumption rate (OCR) of bovine *Psoas major* steaks. Different letters (a–e) indicate significant differences between storage time points for the same age group and packaging method; different letters (x–z) indicate significant differences between packaging methods for the same age group and storage time point (*p* < 0.05).

Mitochondrial activity is an important factor that can influence meat color stability during the postmortem period; mitochondria compete with myoglobin for oxygen, thereby inhibiting the development of bright red OxyMb [32]. Significant effects of harvest age, packaging method, storage time, and their interactions on the OCR value were observed in the present study (*p* < 0.05). Mature cows had a lower initial OCR value (59.3%) compared to younger cattle (64.8%; Figure 4), which may be related to mitochondrial dysfunction in meat from older animals [33]. The OCR was reduced to levels of 35.4, 20.4, and 1.9% for steaks in CO-MAP, PVC, and HiOx-MAP, respectively, on day 14, which was consistent with the findings of Yang et al. [29]. They found that steaks in HiOx-MAP and CO-MAP showed a decreased OCR during storage. CO-MAP showed the highest OCR values for steaks from both young and mature cows during storage compared to aerobic packaging methods. English et al. [10] found that high oxygen environments promote lipid oxidation, resulting in mitochondrial damage and less oxygen consumption. The *Psoas major* muscle has been defined as a very low color stability muscle by McKenna et al. [34]. Recent studies have reported that the decreased color stability of the *Psoas major* muscle can be attributed to greater mitochondrial damage during storage [35,36]. These results indicate that CO-MAP helps preserve mitochondrial functionality and, thereby, oxygen consumption capacity during storage, improving color stability.

### 3.6. Lipid Oxidation

**Figure 5:**
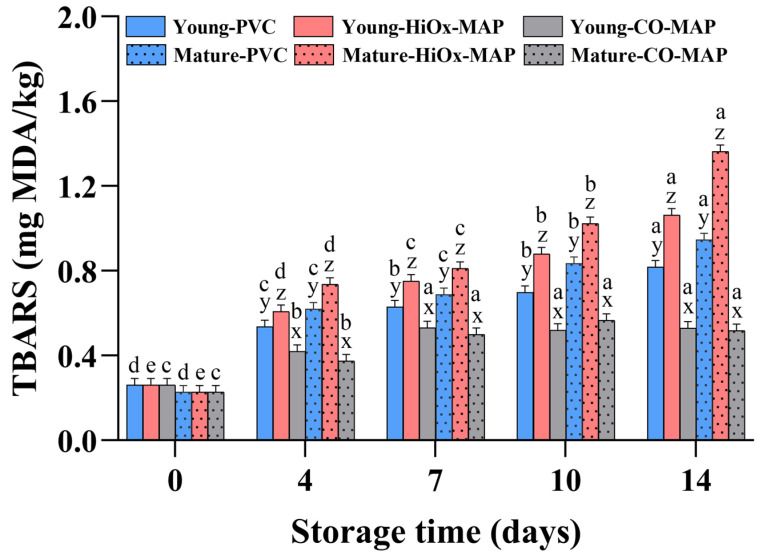
Effect of animal age, packaging method, and storage time on the thiobarbituric acid reactive substance (TBARS) levels of bovine *Psoas major* steaks. Different letters (a–e) indicate significant differences between storage time points for the same age group and packaging method; different letters (x–z) indicate significant differences between packaging methods for the same age group and storage time point (*p* < 0.05).

The results of the TBARS content in young and mature cows under different packaging systems are shown in Figure 5. Significant effects of animal age, packaging method, storage time, and their interactions on TBARS values were found in this study (*p* < 0.05). The TBARS content of both the young and mature cow groups significantly increased (*p* < 0.05) throughout the entire storage period, especially for steaks in the aerobic packages. Among the packaging methods, steaks in HiOx-MAP had the highest TBARS values during storage. Steaks in CO-MAP had the lowest TBARS values at each time point. In aerobic packaging, the mature cow group had higher TBARS levels than the young animals. However, steaks from mature cows exhibited similar TBARS levels to those of young cows in anaerobic CO-MAP. These results are in agreement with the observations reported by Xiong et al. [37] and Cho et al. [5] that the TBARS content increases as the cattle age increases. Increased lipid oxidation in older beef may be due to the higher contents of myoglobin and unsaturated fatty acids [38]. Mitochondrial dysfunction might be another factor related to the higher TBARS value of older cows [33]. Reactive oxygen species produced during aging in aerobic atmospheres may enhance oxidative stress on cell organelles, including mitochondrial membranes, and may also react with meat pigments, stimulating the release of non-heme iron, a known promoter of lipid oxidation [39]. However, there was no significant difference in the TBARS values between young and mature cows in CO-MAP in the current study, likely due to the anaerobic atmosphere in CO-MAP and the high resistance to oxidation of carboxymyoglobin [40,41]. It is worth noting that the TBARS values obtained herein are below 2 mg MDA/kg, the commonly accepted threshold for consumer-perceived rancidity in beef [42]. Overall, lipid oxidation increased with storage time and was more evident in steaks from mature cows and those packaged in aerobic systems, highlighting the combined impact of animal age and packaging on the oxidative stability of meat.

### 3.7. Red Color Penetration Depth

Animal age, package type, and storage time all had significant effects on the red color penetration depth into the beef (*p* < 0.05). Steaks from young cattle showed a thicker surface red band than those from mature animals after 4 days of storage (Table 2). The oxygen penetration depth was deeper for steaks in HiOx-MAP than in PVC at all storage time points, except at day 14 for mature cows. The highest OxyMb thickness was observed for steaks in PVC and HiOx-MAP on day 4 and declined thereafter. Steaks in CO-MAP bloomed slowly, with a general increase in carboxymyoglobin thickness, particularly for young animals. This is in agreement with the findings of Jayasingh et al. [22] and Sakowska et al. [43]. The penetration of the carboxymyoglobin layer was not so great in the first 4 days compared to the thickness of the OxyMb layer in HiOx-MAP, which is likely due to the low CO partial pressure (0.4%) in CO-MAP, compared to 80% O_2_ in HiOx-MAP. Steaks from mature cows had a thicker initial red color layer than their young counterparts, perhaps because of the higher myoglobin content and lower OCR. It is well established that the oxygen penetration depth is inversely related to the OCR in fresh beef steaks exposed to oxygen [34,44]. In the steaks held under PVC and HiOx-MAP, the oxygen penetration depth initially rose and then decreased. The biphasic nature of the oxygen penetration curves is indicative of the initial oxygen penetration and reddening, followed by a phase of gradual pigment oxidation and browning. The subsurface myoglobin forms play an important role in product appearance because MetMb beneath the surface gradually thickens and moves toward the surface. Noticeable surface discoloration occurred in some aerobic packages at 10–14 days of storage, and no surface red band was observed in those samples. The disappearance of the surface oxymyoglobin band might be caused by pronounced lipid oxidation [19]. Monahan et al. [45] stated that oxygen depletion associated with lipid oxidation is a possible factor promoting the oxidation of oxymyoglobin in muscle systems.

## 4. Conclusions

Myoglobin content was higher in *Psoas major* steaks from mature cows compared to those from young animals, resulting in a darker and redder beef color. Regardless of animal age, steaks in PVC and HiOx-MAP had markedly greater discoloration by the end of the 14-day storage period. This is likely due to increased lipid oxidation in aerobic packaging systems compared to anaerobic CO-MAP. Although steaks from both age groups experienced discoloration in aerobic packaging, the effect was more marked in steaks from mature cows. This may be attributed to the greater levels of TBARS and their corresponding effect on impairing the activity of MetMb reductase. In contrast, steaks in anaerobic CO-MAP maintained higher redness, limited discoloration, and lower TBARS values throughout the storage period, with minimal differences between steaks from the two age groups. These observations suggest that both age at harvest and the packaging method influence the shelf stability of *Psoas major* steaks, with the packaging method appearing to be a more important factor. Our results also demonstrate that CO-MAP packaging is more effective than aerobic packaging in preserving the shelf stability of beef and may even mitigate inherent differences in color stability between steaks from young and mature animals.

## Figures and Tables

**Table 1:** Effect of animal age, packaging method, and storage time on the surface color of bovine *Psoas major* steaks.

	Age	Packaging	Storage Time (Days)					
			0	4	7	10	14	SE
L*	Young	PVC	33.2 ^ajx^	32.9 ^ajy^	33.9 ^ajy^	33.8 ^ajy^	33.7 ^ajz^	0.8
		HiOx-MAP	33.2 ^bjx^	40.1 ^ajx^	40.6 ^ajx^	40.4 ^ajx^	38.6 ^ajy^	
		CO-MAP	33.2 ^bjx^	41.5 ^ajx^	42.0 ^ajx^	42.2 ^ajx^	42.5 ^ajx^	
	Mature	PVC	30.2 ^akx^	30.0 ^akz^	30.7 ^aky^	30.9 ^aky^	30.7 ^aky^	
		HiOx-MAP	30.2 ^bkx^	38.7 ^ajx^	37.4 ^akx^	37.7 ^akx^	37.6 ^ajx^	
		CO-MAP	30.2 ^bkx^	36.7 ^aky^	36.0 ^akx^	37.6 ^akx^	37.9 ^akx^	
a*	Young	PVC	13.7 ^akx^	12.7 ^ajy^	10.7 ^bjy^	8.6 ^cjz^	7.8 ^cjz^	0.5
		HiOx-MAP	13.7 ^ckx^	17.3 ^ajx^	16.1 ^abjx^	15.0 ^bcjy^	14.6 ^bcjy^	
		CO-MAP	13.7 ^bkx^	17.3 ^ajx^	17.3 ^akx^	17.5 ^akx^	17.9 ^akx^	
	Mature	PVC	16.1 ^ajx^	12.2 ^bjz^	10.6 ^cjz^	8.8 ^djz^	7.2 ^ejz^	
		HiOx-MAP	16.1 ^ajx^	16.4 ^ajy^	15.6 ^ajy^	13.6 ^bjy^	10.1 ^cky^	
		CO-MAP	16.1 ^bjx^	18.7 ^ajx^	19.8 ^ajx^	19.2 ^ajx^	19.9 ^ajx^	
b*	Young	PVC	13.1 ^abkx^	13.6 ^ajy^	12.8 ^abjy^	12.5 ^abjy^	12.4 ^bjy^	0.4
		HiOx-MAP	13.1 ^ckx^	15.5 ^ajx^	14.8 ^abjx^	14.4 ^bjx^	14.4 ^bjx^	
		CO-MAP	13.1 ^akx^	12.0 ^abjz^	11.8 ^bjy^	12.1 ^abjy^	12.3 ^abjy^	
	Mature	PVC	14.5 ^ajx^	11.9 ^bky^	11.8 ^bjy^	11.1 ^bky^	12.0 ^bjy^	
		HiOx-MAP	14.5 ^ajx^	14.3 ^abkx^	13.1 ^ckx^	13.0 ^ckx^	13.4 ^bcjx^	
		CO-MAP	14.5 ^ajx^	11.8 ^bjy^	12.4 ^bjxy^	12.5 ^bjx^	12.6 ^bjxy^	
h*	Young	PVC	43.6 ^djx^	47.0 ^cjx^	50.0 ^bjx^	55.3 ^ajx^	57.8 ^ajx^	1.1
		HiOx-MAP	43.6 ^ajx^	41.8 ^ajy^	42.8 ^ajy^	43.7 ^ajy^	44.6 ^aky^	
		CO-MAP	43.6 ^ajx^	34.9 ^bjz^	34.3 ^bjz^	34.6 ^bjz^	34.6 ^bjz^	
	Mature	PVC	42.1 ^djx^	44.5 ^djx^	48.1 ^cjx^	51.7 ^bkx^	59.2 ^ajx^	
		HiOx-MAP	42.1 ^bjx^	41.1 ^bjy^	40.2 ^bjy^	43.7 ^bjy^	53.0 ^ajy^	
		CO-MAP	42.1 ^ajx^	32.2 ^bjz^	32.0 ^bjz^	33.1 ^bjz^	32.2 ^bjz^	

Different letters (a–e) indicate significant differences between storage time points for the same row; different letters (j,k) indicate significant differences between age groups for the same time point and packaging method; and different letters (x–z) indicate significant differences between packaging groups for the same time point and age group (*p* < 0.05).

**Table 2:** Effect of animal age, packaging method, and storage time on the red color penetration depth (mm) of bovine *Psoas major* steaks.

Age	Packaging	Storage Time (Days)					
		0	4	7	10	14	SE
Young	PVC	1.02 ^dkx^	2.10 ^ajz^	2.00 ^bjz^	1.81 ^cz^	-	0.02
	HiOx-MAP	1.02 ^ekx^	3.33 ^ajx^	3.03 ^bjx^	2.82 ^cjx^	2.32 ^dy^	
	CO-MAP	1.02 ^dkx^	2.43 ^cjy^	2.42 ^cjy^	2.57 ^bjy^	2.88 ^ajx^	
Mature	PVC	1.15 ^cjx^	1.85 ^akz^	1.57 ^bky^	-	-	
	HiOx-MAP	1.15 ^cjx^	2.38 ^akx^	2.20 ^bkx^	2.20 ^bkx^	-	
	CO-MAP	1.15 ^bjx^	2.18 ^aky^	2.18 ^akx^	2.24 ^akx^	2.20 ^ak^	

Different letters (a–e) indicate significant differences between storage time points for the same row; different letters (j,k) indicate significant differences between age groups for the same time point and packaging method; and different letters (x–z) indicate significant differences between packaging groups for the same time point and age group (*p* < 0.05).

## Data Availability

The original contributions presented in this study are included in the article. Further inquiries can be directed to the corresponding authors.
